# STRAIN AMONG CAREGIVERS OF OLDER BREAST CANCER PATIENTS

**DOI:** 10.1093/geroni/igz038.2388

**Published:** 2019-11-08

**Authors:** Janine A Overcash, Susan Fugett, Alai Tan, Jane Ginther, Nicole Williams

**Affiliations:** 1 The Ohio State University, College of Nursing, Columbus, Ohio, United States; 2 Stephanie Spielman Comprehensive Breast Center, The Ohio State University, Columbus, Ohio, United States; 3 The Ohio State University, Columbus, Ohio, United States

## Abstract

Caregiver strain is the emotional and physical demands associated with providing care, particularly when the needs exceed the resources. The purpose of this presentation is to illustrate the predictors of strain among caregivers of older breast cancer patients. Relationships among caregiver strain (Modified Caregiver Strain Index), age, employment status, patient characteristics and scores on the comprehensive geriatric assessment (grip strength, Timed up & Go, Mini Nutritional Assessment, MiniCog, Geriatric Depression Scale, Pittsburgh Sleep Quality Index, and Brief Fatigue Inventory) are described. This cross sectional study included women who were diagnosed with breast cancer, over the age of 69 years, receiving any type of treatment and seeking an initial assessment in a geriatric oncology program.. Dyads (N=39) had a mean patient age of 77 and caregiver age of 64 years. Most patients were diagnosed with infiltrating ductal carcinoma 30 (76.9%), most had lumpectomy 23 (59%), and were stage 1 15 (38.5%). Most patients had one caregiver 22(56.4%) who worked full-time 12(30.8) and rated their health as very good 16 (25.4%). Increasing age of the caregiver was associated with less caregiver strain (r = -0.45, p=.02). Employment status of the caregiver was significantly related to caregiver strain -0.35 (p=.03). Caregivers employed full time 3.5 (p = .08), and part time 5.9 (p=0.3) experienced greater strain (mean±SD: 9.4±8.8) than those employed full-time (4.7±3.4). Patient depression, caregiver employment status and age of the caregiver are related to caregiver strain. Healthcare providers must assess for strain in cancer givers of cancer patients.

